# Supplementary material for: Electrically induced and detected Néel vector reversal in a collinear antiferromagnet
Source: Nat Commun. 2018 Nov 8;9:4686. doi: 10.1038/s41467-018-07092-2 (PMC6224378; doi:10.1038/s41467-018-07092-2)
Supplement: Supplementary file 1 — Supplementary Information [file 41467_2018_7092_MOESM1_ESM.pdf]

Supplementary information: Electrically induced and detected Néel vector reversal in a collinear antiferromagnet

J. Godinho,[1,2] H. Reichlová,[1,3] D. Kriegner,[1,4] V. Novák,[1] K. Olejník,[1] Z. Kašpar,[1] Z. Šobáň,[1] P Wadley,[5] R. P. Campion,[5] R. M. Otxoa,[6,7] P. E. Roy,[6] J. Železný,[1] T. Jungwirth,[1,5] and J. Wunderlich[1,6]

[1]*Institute of Physics ASCR, v.v.i., Cukrovarnická 10, 162 53, Praha 6, Czech Republic*

[2]*Faculty of Mathematics and Physics, Charles University in Prague, Ke Karlovu 3, 121 16 Prague 2, Czech Republic*

[3]*Institut für Festkörper- und Materialphysik, Technische Universität Dresden, 01062 Dresden, Germany*

[4]*Max Planck Institute for Chemical Physics of Solids, 01187 Dresden, Germany*

[5]*School of Physics and Astronomy, University of Nottingham, Nottingham NG7 2RD, United Kingdom*

[6]*Hitachi Cambridge Laboratory, Cambridge CB3 0HE, United Kingdom*

[7]*Donostia International Physics Center, Paseo Manuel de Lardizabal 4, Donostia-San Sebastian 20018, Spain*

SUPPLEMENTARY NOTE 1. DEVICE FABRICATION AND MATERIAL CHARACTERIZATION

Superconducting quantum interference device (SQUID) magnetometry of our material is shown in Supplementary figure 1a. X-Ray diffraction (XRD) measurements are shown in Supplementary figure 1b.

SUPPLEMENTARY NOTE 2. EXPERIMENTAL TECHNIQUES

A. Electrically induced Néel vector deflection combined with AMR

We first describe in more detail the microscopic mechanism of the second order magnetoresistance in which electrically induced Néel vector deflection is combined with AMR. The current-induced staggered spin-polarization (Supplementary figure 2a) generates spin-orbit fields $\mathbf{B}^{\mathbf{A}}_{\mathbf{SO}}$ and $\mathbf{B}^{\mathbf{B}}_{\mathbf{SO}}$, with $\mathbf{B}^{\mathbf{B}}_{\mathbf{SO}} = -\mathbf{B}^{\mathbf{A}}_{\mathbf{SO}}$, acting on the corresponding sublattice magnetizations $\mathbf{M_A}$ and $\mathbf{M_B}$ of the bipartite antiferromagnet CuMnAs. $\mathbf{B}^{\mathbf{A,B}}_{\mathbf{SO}}$ are oriented perpendicular to the applied current direction and their magnitude is proportional to the applied current density j. In equilibrium, $\mathbf{M_A} = -\mathbf{M_B}$, so that the corresponding spin-orbit torques $\mathbf{B}^{\mathbf{A}}_{\mathbf{SO}} \times \mathbf{M_A} = \mathbf{B}^{\mathbf{B}}_{\mathbf{SO}} \times \mathbf{M_B}$ cant the sublattice magnetisations from their antiparallel equilibrium orientation. The resulting exchange torques then rotate the sublattice magnetisations $\mathbf{M_{A,B}}$ within the basal plane of CuMnAs towards the direction of the spin-orbit fields $\mathbf{B}^{\mathbf{A,B}}_{\mathbf{SO}}$. Our detection method is based on the fact that the spin-orbit-torques and the resulting exchange torques flip their signs when the sublattice magnetisations reverse and therefore deflect the reversed Néel vector in the opposite direction (see Supplementary figs. 2b,c). This combined with AMR makes the second order magneto-resistance, in general, unequal for the reversed states and allows for the electrical detection of the Néel vector reversal.

At high-amplitude setting current pulses the antiferromagnetic moments are aligned with the direction of the current-induced spin-orbit fields[1,2]. At low probing currents (weak spin-orbit fields relative to anisotropy fields), the antiferromagnetic moments are only deflected by a small angle $\delta\varphi$ proportional to the magnitude of the current induced spin-orbit fields. This combined with the AMR results in a second-order magneto-transport effect and a corresponding resistance variation, δR_{ij}, that depends linearly on the probing current. To de-

scribe the φ-dependence of δR_{ij} we first recall the angular dependence of the linear-response AMR. Assuming that AMR in CuMnAs is dominated by the non-crystalline component, the longitudinal AMR is given by $R_{xx} = R_0 + \Delta_{\mathrm{AMR}} \cdot \cos(2\varphi)$ and the transverse AMR by $R_{xy} = \Delta_{\mathrm{AMR}} \cdot \sin(2\varphi)$, with $\Delta_{\mathrm{AMR}} = \frac{1}{2}[R_{xx}(\mathbf{M_{A,B}} \parallel \mathbf{j}) - R_{xx}(\mathbf{M_{A,B}} \perp \mathbf{j})]$.

Supplementary fig. 2b shows a scenario where in one panel the equilibrium Néel vector is set at an angle $\varphi = 45°$ from the x-axis of the reading current while in the other panel the equilibrium Néel vector is reversed. When the current j is applied, the antiferromagnetic moments are deflected clockwise by $-\delta\varphi$ or counter-clockwise by $+\delta\varphi$ depending on the equilibrium Néel vector direction. The longitudinal resistance of CuMnAs then decreases or increases by δR_{xx} due to the longitudinal AMR. In Supplementary fig. 2c, we sketch the scenario where the Néel vector is aligned with the x-axis of the reading current. In this configuration, the current induced Néel vector deflection results in the transversal resistance variation $\pm\delta R_{xy}$, depending on the direction of the Néel vector. Since δR_{xx} and δR_{xy} are current depend, we call them nonlinear AMR contributions in contrast to the current independent R_{xx} and R_{xy} which we call linear AMR contributions.

The easy plane magnetic anisotropy of our CuMnAs crossbar devices enabled us to set the Néel vector along a series of different in-plane directions (we measured 8 directions). With this we could perform extensive consistency checks between the signs of the linear and nonlinear AMR contributions measured in both longitudinal and transverse geometries. The results are in full agreement with the scenario of the second-order magnetoresistance that combines the current-induced Néel vector deflection with the AMR. We note that these consistency checks did not require the knowledge of the sign of the staggered current induced spin-orbit field on a given spin-subblatice for a given current direction. This is because in our measurements of δR_{xx} and δR_{xy}, the sign enters twice: first, when set the Néel vector direction by the staggered spin-orbit field and, second, when we detect the Néel vector direction via the staggered spin-orbit field deflection of the Néel vector.

B. Detection of the nonlinear AMR

In order to separate the linear and nonlinear AMR contributions, we apply an alternating probing current $J_0 \sin(\omega t)$ along the x-axis (corresponding to a low current density $\sim 1 \times 10^6$A cm^{-2}) of frequency $\omega/2\pi = 143$ Hz.

At such a quasi-static condition, assuming that the spin-orbit field is rotated clockwise (anti-clockwise) with respect to the current direction, the deflection angle $\delta\varphi \sim -(+)J_0\cos(\varphi)\sin(\omega t)$ and the corresponding longitudinal and transversal resistance variations $\delta R_{xx}(\varphi,t) = \frac{\partial R_{xx}}{\partial\varphi}\cdot\delta\varphi$ and $\delta R_{xy}(\varphi,t) = \frac{\partial R_{xy}}{\partial\varphi}\cdot\delta\varphi$ follow directly the alternating current without phase-shift, so that

$$\delta R_{xx}(\varphi,t) \sim +(-)2J_0\cdot\Delta_{\mathrm{AMR}}\cdot\cos(\varphi)\cdot\sin(2\varphi)\cdot\sin(\omega t) \text{ and}$$

$$\delta R_{xy}(\varphi,t) \sim -(+)2J_0\cdot\Delta_{\mathrm{AMR}}\cdot\cos(\varphi)\cdot\cos(2\varphi)\cdot\sin(\omega t).$$

Since both ac-current and device resistance oscillate at the same frequency ω, Ohm's law yields, $\delta V_{xx} = \delta R_{xx}(\varphi,t)\cdot J_{ac}(t) \sim J_0\cdot\Delta_{\mathrm{AMR}}\cdot\cos(\varphi)\cdot\sin(2\varphi)\cdot(1+\sin(2\omega t-90^\circ))$ and $\delta V_{xy} = \delta R_{xy}(\varphi,t)\cdot J_{ac}(t) \sim -J_0\cdot\Delta_{\mathrm{AMR}}\cdot\cos(\varphi)\cdot\cos(2\varphi)\cdot(1+\sin(2\omega t-90^\circ))$. Therefore, the nonlinear AMR appears only as a time-independent constant voltage and as a second harmonic voltage signal oscillating at twice of the alternating reading current frequency.

In our experiments we use lock-in amplifiers to measure simultaneously longitudinal and transversal voltage signals at the current frequency ω (first harmonic signals $V_{xx}^{1\omega}$ and $V_{xy}^{1\omega}$) and at twice of the current frequency 2ω (second harmonic signals $V_{xx}^{2\omega}$ and $V_{xy}^{2\omega}$). The first harmonic signals contain only the linear AMR responses since the contributions from the nonlinear AMR average out to zero. From the second harmonics signal we can exclude contributions from the Joule heating since they do not depend on the Néel vector orientation and a possible contribution from the magneto-thermopower is an even function under Néel vector reversal and also small in our symmetric devices. Contributions from the anomalous Nernst effect do not appear in antiferromagnetic CuMnAs for the same symmetry reason (PT-symmetry) as discussed in the main text in the context of the absence of the anomalous Hall effect. We therefore can assign linear and non-linear AMR to the measured signals as

$$R_{xx}^{1\omega}(\varphi) = \mathrm{Re}(V_{xx}^{1\omega})(\Delta\phi = 0^\circ)/J_0 = R_0 + \Delta_{\mathrm{AMR}}\cdot\cos(2\varphi),$$

$$R_{xy}^{1\omega}(\varphi) = \mathrm{Re}(V_{xy}^{1\omega})(\Delta\phi = 0^\circ)/J_0 = \Delta_{\mathrm{AMR}}\cdot\sin(2\varphi),$$

$$R_{xx}^{2\omega}(\varphi) = \mathrm{Re}(V_{xx}^{2\omega})(\Delta\phi = -90^\circ)/J_0 \sim \Delta_{\mathrm{AMR}}\cdot\cos(\varphi)\cdot\sin(2\varphi), \text{ and}$$

$$R_{xy}^{2\omega}(\varphi) = \mathrm{Re}(V_{xy}^{2\omega})(\Delta\phi = -90^\circ)/J_0 \sim -\Delta_{\mathrm{AMR}}\cdot\cos(\varphi)\cdot\cos(2\varphi),$$

where $\mathrm{Re}(V)$ is the part of the measured signal detected by the lock-in amplifiers which oscillates delayed by the phase-shift $\Delta\phi$ with respect to the reading current.

SUPPLEMENTARY NOTE 3. EFFECT OF CAPPING LAYERS ON SWITCHING PROPERTIES OF THE DEVICES

To evaluate the effect of the 3 nm Pt layer on top of the 10 nm CuMnAs layer, a reference film was grown simultaneously by masking part of the wafer during Pt evaporation. Supplementary figure 3 shows the bipolar switching characteristics of a 4-contact cross-bar device with 10 μm wide bars patterned from the reference CuMnAs/AlOx film without the Pt-layer. Here we measured the transverse second-harmonic resistance $R_{xy}^{2\omega}$ as a response to the probing ac-current of effective value $J_{ac} = J_0/\sqrt{2} = 1$ mA ($j_{ac} \sim 1 \times 10^6$ A cm^{-2}) applied along the x-axis after 20 ms long, 9 mA writing pulses ($j_{ac} \sim 9 \times 10^6$ A cm^{-2}) applied along the y-axis. The measured $R_{xy}^{2\omega}$ shows again the expected dependence of the second harmonics signal on the polarity of the setting current pulses corresponding to reversed Néel vector states. Note that in this reference sample, setting current pulses of a $\sim 30\%$ higher current density were required. We assign the difference in required switching current densities to the difference in Joule heating between the devices patterned from the CuMnAs/Pt/AlOx film and the devices patterned from the CuMnAs/AlOx film without Pt.

The total sheet resistance R_T of the CuMnAs(10nm)/Pt(3nm)/AlOx stack is $\sim 100\ \Omega$, which is approximately 4× lower than the sheet resistance of the refernce CuMnAs(10nm)/AlOx film. Therefore, in the stack containing the Pt layer, only 1/4-th of the total applied current flows through the CuMnAs layer and 3/4-th of the current flow through the highly conductive Pt layer, which increases the sample temperature during the setting current pulse and facilitates the current induced switching. Note that the Joule heating in the film containing Pt at the same current density in the CuMnAs layer is about 4× larger than in the reference CuMnAs/AlOx film, since $R_{\mathrm{Pt}} \cdot I_{\mathrm{Pt}}^2 + R_{\mathrm{CuMnAs}} \cdot I_{\mathrm{CuMnAs}}^2 = 1/3 R_{\mathrm{CuMnAs}} \cdot (3 \cdot I_{\mathrm{CuMnAs}})^2 + R_{\mathrm{CuMnAs}} \cdot I_{\mathrm{CuMnAs}}^2 = 4 R_{\mathrm{CuMnAs}} \cdot I_{\mathrm{CuMnAs}}^2$.

Apart from Joule heating, an additional spin-orbit torque generated by the current flowing through the CuMnAs/Pt interface could be considered to affect magnetisation dynamics[1,4]. This torque can originate from the spin Hall effect in Pt or from the inverse spin galvanic (Edelstein) effect at the CuMnAs/Pt interface. Both effects would result in a non-staggered interfacial spin-polarisation $\mathbf{p}$ oriented along the y-axis when the current flows along the x-axis. In this case, the antidamping-like torque, which is driven by the sub-lattice magnetisation dependent staggered antidamping fields, $\sim \mathbf{p} \times \mathbf{M_A}$ and $\sim \mathbf{p} \times \mathbf{M_B} = -\mathbf{p} \times \mathbf{M_A}$, can,

in principle, efficiently act on the antiferromagnetic state[1,4]. However, in case of CuMnAs, this interfacial spin-orbit torque remains inefficient. It cants the sub-lattice magnetisations towards the in-plane orientation perpendicular to the applied current direction and the resulting exchange torques would then trigger Néel vector reorientation towards the out-of-plane direction. This is inefficient, however, due to the strong, out-of-plane hard-axis anisotropy in tetragonal CuMnAs.

Supplementary References

1 Železný, J. *et al.* Relativistic Néel-Order Fields Induced by Electrical Current in Antiferromagnets. *Physical Review Letters* **113**, 157201 (2014).

2 Wadley, P. *et al.* Electrical switching of an antiferromagnet. *Science* **351**, 587–590 (2016).

3 Wadley, P. *et al.* Tetragonal phase of epitaxial room-temperature antiferromagnet CuMnAs. *Nature Communications* **4**, 2322 (2013).

4 Jungwirth, T., Marti, X., Wadley, P. & Wunderlich, J. Antiferromagnetic spintronics. *Nature Nanotechnology* **11**, 231–241 (2016).

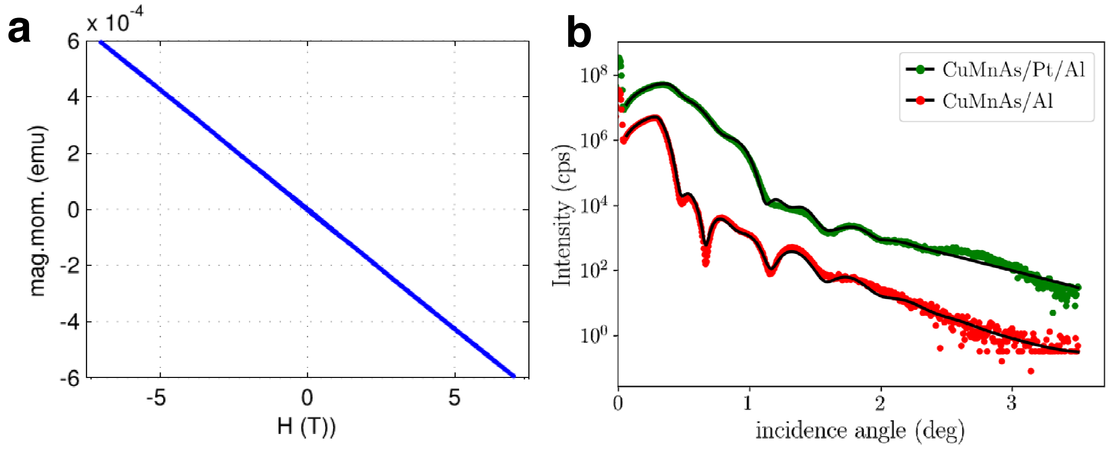

Supplementary Figure 1. (a) SQUID measurement on a CuMnAs(10nm)/Pt(3.6nm)/Al(4nm) film. Magnetization loop up to 7 T shows no indication of a net magnetic moment. (b) X-ray reflectivity measurement on the CuMnAs(10 nm)/Pt(3.6 nm)/Al(4 nm) film. The panel shows the angular dependence of the reflection signal for CuK_α radiation at grazing angles. Coloured points show the experimental data while solid lines show our model calculation based on the Parrat formalism. Data for the sample with Pt were scaled by a factor of 10 for clarity.

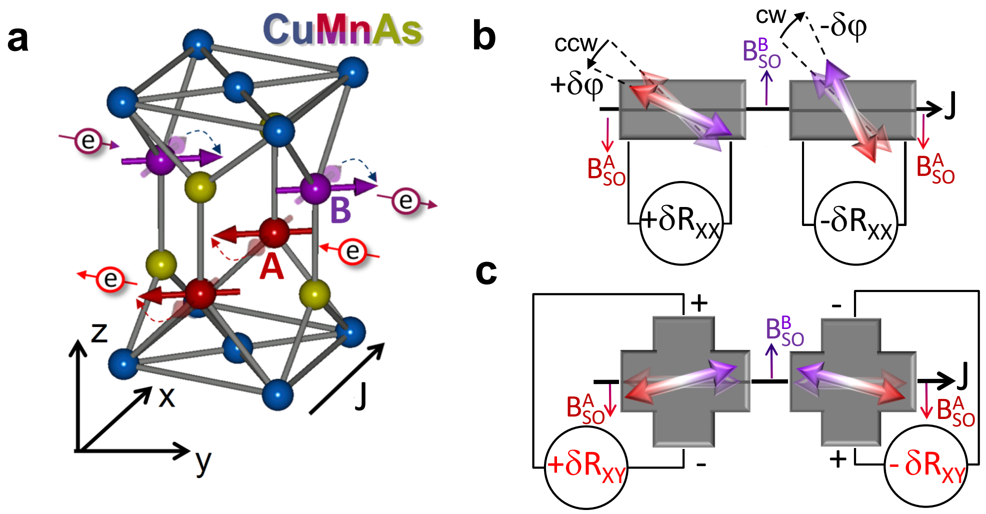

Supplementary Figure 2. (a) Antiferromagnetic CuMnAs with collinear spin-sublattices A and B. The two sites occupied by nearest neighbour Mn atoms are locally non-centrosymmetric inversion partners and belong to opposite spin-sublattices of the bipartite Néel order ground state. When biased by a charge-current J, a staggered spin-polarisation perpendicular to the current direction is generated with opposite sign on the two sites. The antiferromagnetic moments rotate towards the staggered spin polarisation. (b) Clockwise (cw) and counter-clockwise (ccw) rotation of the sublattice magnetisations for reversed Néel vector states. Corresponding current dependent longitudinal resistance variation $\pm\delta R_{xx}$ for the Néel vector oriented at an angle $\varphi = 45°$ from the probing current axis. (c) Current dependent transverse resistance variation $\pm\delta R_{xy}$ for the Néel vector oriented along the probing current axis.

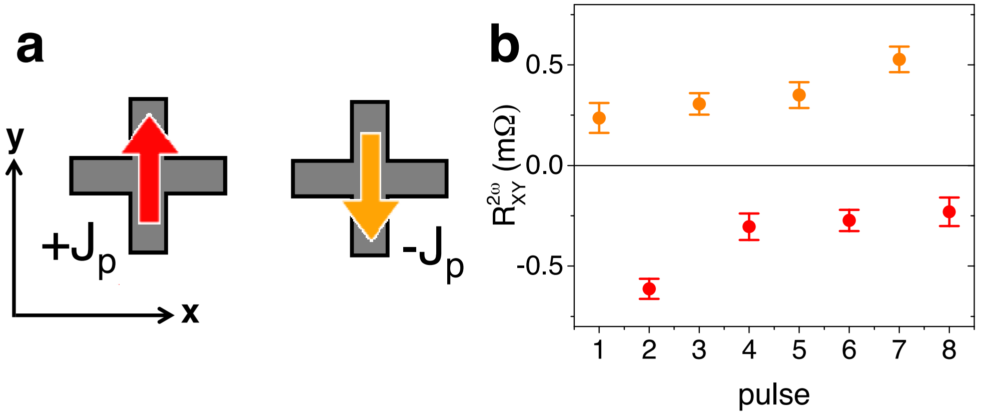

Supplementary Figure 3. (a) Measurement set up with writing pulses along $+y$ (red) and $-y$ (orange) directions in a 10 μm wide 4 terminal cross bar device patterned from a reference CuMnAs(10nm)/AlOx film without Pt layer. The writing pulse amplitude $J_p = 9$ mA, the pulse duration $\tau_p = 20$ ms. (b) Second-harmonic transverse resistance $R_{xy}^{2\omega}$ with probing current of $J_{ac} = 1$ mA. along x-axis.
